# Social identities and mental well-being in autistic adults

**DOI:** 10.1177/13623613211004328

**Published:** 2021-05-19

**Authors:** Cameron A Maitland, Sinead Rhodes, Anne O’Hare, Mary E Stewart

**Affiliations:** 1 University of Edinburgh, UK; 2 Heriot-Watt University, UK

**Keywords:** adults, autism spectrum disorders, depression, mental health, social cognition and social behaviour, social identity, well-being

## Abstract

Social identities relate to psychological perceptions of group memberships and form part of the self-concept. Socially identifying with groups has previously been found to associate with better mental well-being outcomes. This study first examined the factor structure and the reliability of measuring social identification in autistic adults. Confirmatory factor analysis showed that a factor structure was replicated in this sample for social identification with other autistic people, but not the family. Second, the study assessed the level to which autistic adults socially identified with different groups, the total number of social identities and whether these were associated with their mental well-being. Autistic adults reported feelings of social identification with many kinds of groups, some with multiple groups, whereas others did not socially identify with any group. Stronger feelings of social identification towards other autistic people and towards one’s family, and with more groups overall, were associated with less severe self-reported depression symptoms and more facets of positive mental health. These findings indicate the importance of facilitating autistic people’s engagement with social groups.

**Lay abstract:**

Social identities are groups that we are part of and influence how we think about ourselves. However, up until now there has been little examination of the groups that autistic people may belong to, and how these groups may influence their mental health. This survey-based study investigated whether autistic adults answer questions about social groups in a similar way to non-autistic non-autistic adults, including the types and number of social groups they may belong to, and whether these are associated with depression, anxiety and positive traits of mental well-being. In total, 184 autistic adults completed an online survey with questionnaires about their demographics, social groups and mental health. The results found that autistic adults reported on their social groups similarly to non-autistic people. There was a variety in the types and numbers of groups that autistic adults identified with. Some participants reported having no groups that they identified with, whereas others reported up to four groups. These included other autistic people, their family, friends, work colleagues and activity clubs among others. Autistic adults who felt connected with more groups reported better mental well-being. Feelings of connection to other autistic people and the family were also associated with better mental well-being. These results show that it is important for autistic people to be given opportunity to be part of groups that are meaningful to them, as this may be beneficial for their mental health.

Autistic people are generally considered to be more vulnerable to poor mental well-being than those without neurodevelopmental conditions and are at greater risk of having a mental health condition (Lai et al., 2019) and generally poorer psychological health (Kamio et al., 2013; Kamp-Becker et al., 2010). A recent meta-analysis showed estimated rates of current depressive disorders in autistic child and adult samples of 11%, compared to 4.7% in general population samples (Lai et al., 2019). This meta-analysis also found anxiety disorders were more common in the autistic population (20% compared to 7.3%). These statistics succinctly summarise a predominant trend of autistic people being at higher risk of mental illness. The concept of positive mental health comprises both feeling and functioning well (Stewart-Brown et al., 2009) and as constituting more than just a lack of mental illness (World Health Organization, 2004). The academic literature is inconclusive regarding a clear distinction between mental health and well-being. Positive mental health and mental well-being are terms that are used interchangeably (Tennant et al., 2007). Here, we use mental well-being as an overarching phrase to encapsulate the full spectrum of mental health, from specific negative symptom patterns of anxiety and depression to positive markers, such as feeling optimistic and able to cope. We use positive mental health to specifically describe markers of this positive end of the scale of mental well-being.

In one study that reported positive mental health scores for an autistic adult sample (Hedley et al., 2019), scores were lower than the median and outside the interquartile range of scores of the general population (Tennant et al., 2007). Though there was no statistical analysis to examine whether this was a significant difference, it suggests that autistic adults have less positive mental health than the neurotypical population. There is a need to identify factors which associate with the risk of poorer mental well-being in autistic people, in both the form of mental ill health symptoms, such as depression and anxiety, and levels of positive mental health.

The ‘social cure’ research agenda proffers the idea that the groups we consider ourselves part of and are important to us have substantial impacts on health (Jetten et al., 2017). The social identity approach (SIA; Jetten et al., 2017) is based on two theories. Social identity theory (Tajfel & Turner, 1979) posits that one’s identity can be considered in two ways. Our *personal identity* comprises those characteristics which mark us as different to anyone else within a group. Our *social identity* comprises the attributes that we share with others in a group and demarks how that group of people is different to other groups. Self-categorisation theory (Turner et al., 1994) proposes that when a group membership is salient to an individual, they come to perceive themselves more in terms of the shared attributes, norms and aspirations of the group, rather than the personal identity characteristics which mark them as distinct from others. By doing so, the individual’s self-concept is altered to become more similar to that of the group with which they identify. Social identities are therefore people’s psychological group memberships, as reflected in their self-concept.

The SIA gives two ways to consider social identity. First, one may select a specific group and measure the *degree* to which individuals feel connection and belonging to that group. Second, an individual may have multiple groups to which they feel connected and which influence their self-perceptions. Both have been associated with better mental health in non-autistic samples. Stronger feelings of social identification (SI) have been associated with lower levels of depression (Postmes et al., 2019) and anxiety (Wakefield et al., 2013) and higher levels of positive mental health (Williams et al., 2019). A meta-analysis of interventions aimed at increasing SI found consistent improvements in depression, anxiety and positive mental health across samples, and that increases in SI were an important mechanism for these effects (Steffens et al., 2019). Although in some circumstances, stronger SI may be associated with poorer well-being. Perceived incompatibility between an existing and a potential social identity may hinder the development of SI with a new group and negatively impact adapting to life transitions (Iyer et al., 2009). Socially identifying with a group with unhealthy norms of behaviour may also be harmful. For example, depressed individuals who more strongly identified with a depressed social identity and had clearer ideas of the normative behaviour associated with being a depressed person showed poorer mental well-being (Cruwys & Gunaseelan, 2016). As such, SI with a group tends to be associated with benefits for mental well-being, but certain social identities may be harmful.

Considering the number of groups people socially identify with, Sani et al. (2015b) found that 44.6% of those with no social identities were classed as depressed, based on scoring above cut-off on the self-report Major Depression Inventory (Cuijpers et al., 2007). Of those socially identifying with one group, 17.1% were classed as depressed. However, 5.7% and 2.4% of those reporting three and four groups, respectively, were depressed. The differences in the proportions classed as depressed were significant. Similar studies have found that having more social identities is significantly related to lower levels of general psychological distress (Cientanni et al., 2017; Miller, Wakefield & Sani., 2017).

It is not yet known whether social identities are associated with mental well-being in autistic people. Before such associations can be explored, it must first be established that SI can be measured accurately in autistic people. It has been reported that autistic individuals can find it difficult to reflect on their own internal feelings (Happé, 2003; Hill et al., 2004) and may understand and respond to questions differently to non-autistic individuals (Happé, 1995). It is therefore important to test whether existing SI scales are valid and reliable for autistic people. The Integrated Self-Categorisation model of Autism (ISCA) posits that autistic people’s general bias towards local over global processing and categorisation (Mottron et al., 2006) extends to the self-categorisation process which underlies SI (Bertschy et al., 2020). When presented with the need to self-categorise, this can mean that idiosyncratic traits of personal identity are more salient to an autistic person, rather than their shared characteristics with members of a group. This decreased likelihood of global self-categorisation may consequently mean a decreased tendency for group-based SI. Direct evidence for the ISCA has only been observed in relation to the degree of autistic traits, rather than specifically comparing autistic and non-autistic individuals. However, relatively lower levels of SI in autistic people have been observed. For instance, autistic adults have reported lower SI with gender groups than non-autistic adults have (Cooper et al., 2018). SI may therefore be experienced differently by autistic people.

Qualitative studies have revealed that many autistic people report feeling ‘different’ and as if they do not ‘fit in’ with others, leading to feelings of isolation and inferiority (e.g. Humphrey & Lewis, 2008; Milton & Sims, 2016). Loneliness is considered as the painful feeling that arises from a discrepancy between the social contact and relationships that a person perceives themselves having and the social relationships that they desire (Peplau & Perlman, 1982). Loneliness has been associated with higher depression in autistic adolescents (Whitehouse et al., 2009) and autistic adults (Hedley et al., 2018; Mazurek, 2014). In the general population, loneliness has strong evidential support as a predictor of depression (Cacioppo et al., 2006; Jaremka et al., 2014; Luo et al., 2012). For example, it has been found to longitudinally predict depression symptoms while controlling for objective social network size, and with the reverse direction of depression predicting loneliness being non-significant (Cacioppo et al., 2010). Although conceptually similar in that both relate to feelings of social connection, loneliness indicates a general sense of disconnection from others, whereas SI as a variable relates to feelings of similarity and belonging with a specific group. As such, this means that social identities provide specific targets for decreasing loneliness (and the depression associated with it), and that improving feelings of connection with specific groups will diminish an overall sense of loneliness. Indeed, increases in SI have been associated with decreases in loneliness (Haslam et al., 2016).

Cooper et al. (2017) asked adult autistic participants about their SI with other autistic people. Path analyses showed that greater feelings of SI predicted greater collective self-esteem, which concerns how positively participants felt about autistic people as a social group and how positively they felt others perceived autistic people. Higher collective self-esteem was in turn associated with higher personal self-esteem. With these self-esteem variables as mediators, SI was indirectly associated with both anxiety and depression. This illustrates that feelings towards other autistic people can influence an autistic individual’s perceptions of themselves and mental well-being. Although not a direct investigation of SI, recent qualitative evidence has suggested links between autistic people’s sense of belonging with each other and well-being (Crompton et al., 2020). Autistic adults reported that being able to spend time with other autistic people provided a sense of belonging. They felt that other autistic people were able to understand them and they were able to be their true authentic self. This was seen by participants as being important for maintaining mental well-being.

## Current study research questions

Autistic people’s group relationships may be influential on their mental well-being, and therefore their social identities warrant exploring. It was first necessary to investigate whether autistic people experience and report on their social identities in the same or different ways to the rest of the population. This study therefore examined whether an existing SI measure has internal reliability and demonstrates the same factor structure in an autistic sample as in general population samples. In addition, SI in autistic people has been examined for relatively few groups, namely autistic social identity (Cooper et al., 2017) and gender groups (Cooper et al., 2018). There are countless other social identities which may also be important. This study asked autistic adults about the different groups they are part of, and their feelings of SI towards them. Although SI has been associated with depression and anxiety in autistic adults, it has not been shown whether it also relates to their levels of positive mental health. For a more rounded perspective on autistic mental well-being, this study examined whether SI associates with each of depression, anxiety and positive mental health.

## Methods

### Public involvement

The researchers sought the autistic community’s opinions on an original study proposal to examine social identities and mental well-being. An online visual presentation was created, outlining social identity theory and the planned study. This presentation was distributed through C.A.M.’s Twitter account. Autistic individuals were invited to provide opinion and feedback on the perceived value and applicability of social identity theory and the proposed study. Feedback suggested that in general, the autistic community considered the planned research to be worthwhile. Changes were made to original methodology in line with feedback. These changes were to allow self-diagnosed autistic adults to participate rather than just those with a formal diagnosis, to measure anxiety and positive facets of mental health rather than solely depression and to specifically examine the autistic social identity. The researchers thank all those who contributed and hope that this study is of greater value to the autism community as a result.

### Participants

Ethical approval was granted by the Heriot-Watt School of Social Sciences Ethics Committee. The study recruited adults aged 18 years or older, who had English as a first language and who considered themselves autistic, whether clinically diagnosed or self-identifying as autistic. Participants were recruited through posters advertising the study placed around two universities and local businesses and venues. Disability services of UK universities were contacted and asked to display posters. The study was circulated through the first author’s Twitter account. The autism research organisation Autistica distributed an advertisement for the study through their Discover network, a network of individuals from the autism community who take an active interest in autism research. Other autism organizations, including Scottish Autism and Autism Initiatives, also distributed an advert for the study through their services and communication platforms.

The average correlation coefficient between SI and depression across 14 previous studies was −0.33 (Cruwys et al., 2014). Using Cohen’s (1988) tables, 160 participants are needed to detect an r of 0.20 at power 0.80 at α = 0.05. In total, 184 participants were recruited: 160 individuals (87%) reported having a formal autism diagnosis and 24 individuals (13%) reported self-diagnosing as autistic. Of the total 184, 147 met the cut-off on the Social Responsiveness Scale–Second Edition (SRS-2; Constantino & Gruber, 2012). All the 37 participants who did not meet the cut-off reported a formal autism diagnosis. This resulted in all 184 participants being included as part of the analytic sample. Demographics are shown in Table 1. Of the total 184, 58 (31.5%) were male, 115 (62.5%) were female and 11 (6%) were of other gender. Ages ranged from 18 to 67 (*M* = 41.0, *SD* = 12.8) years.

**Table 1.** Participant demographics (N = 184).

Demographic variable	n	%
Gender		
Male	58	31.5
Female	115	62.5
Other	11	6
Employment type		
Full time	66	35.9
Part time	28	15.2
Self-employed	14	7.6
Unemployed	13	7.1
Unable to work	20	10.9
Retired	11	6
Student	24	13
Career	7	3.8
Prefer not to say	1	0.5
Ethnic group		
White	171	92.9
Mixed/multiple ethnic groups	7	3.8
Asian/Asian British	1	0.5
Black/African/Caribbean/Black British	1	0.5
Other ethnic group	2	1.1
Prefer not to say	2	1.1
Highest educational attainment		
Post-degree	22	12
Undergraduate/masters degree	102	55.4
Post-secondary	30	16.3
Upper secondary	17	9.2
Lower secondary	8	4.3
Primary education	1	0.5
Less/none	4	2.2
Relationship status		
Married/long-term partner	87	47.3
In a relationship	17	9.2
Single	70	38
Divorced/separated	9	4.9
Prefer not to say	1	0.5

### Measures

#### SRS-2 – Adult Self-Report Version

The SRS-2 assesses an individual’s autism-related social functioning (Constantino & Gruber, 2012). Across 65 items, an individual rates the frequency with which they observe a behaviour in themselves on a 4-point scale (1 = Not True, 2 = Sometimes True, 3 = Often True, 4 = Always True). Scores are then converted to T-scores, with higher scores indicating more self-perceived autistic social behaviours. The cut-off T-score of 60 indicating mild autistic symptoms was applied as an inclusion criterion for self-diagnosed participants only.

#### SI

Leach et al. (2008) constructed a 14-item measure for assessing SI based on an evaluation of previous SI instruments. Items are categorised into five different components of SI: individual self-stereotyping, in-group homogeneity, satisfaction, solidarity and centrality. These load onto two separate higher order factors; the first two components comprise the self-definition factor and the latter three components comprise the self-investment factor in a hierarchical model. Participants rate their agreement with a statement relating to their feelings towards a specified group on a 7-point scale (1 = Strongly Disagree, 7 = Strongly Agree). Overall scores for a given group can range from 14 to 98.

Items of the scale can be altered for specific groups. Participants first completed the measure with autistic people specified as the in-group (e.g. ‘I feel a bond with autistic people’ and ‘Being autistic is an important part of how I see myself’), giving an Autism social identification (Autism SI) score. Second, participants completed the measure with their family specified as the in-group (Family SI score). Participants were then asked to consider the group of people they spend the most face-to-face time with during a week who are not their family. They named the group, indicated the nature of the group (e.g. classmates, friends, colleagues) and approximately how many hours per week they spend with the group. They then completed the SI measure for this group, giving a Contact group social identification (Contact SI) score. Having completed this measure three times and gained familiarity with the questions, participants were asked to consider whether they had another group which the item statements applied to. Again, participants named the group and indicated its nature and approximate weekly hours spent interacting with. They then answered the SI items referencing this group, giving a self-nominated group social identification (Self-Nom SI) score. Participants were instructed not to respond to the Contact SI or Self-Nom SI questions if they did not feel they had such a group. If participants described and answered about a group that did not match the current study’s concept of a group (e.g. an individual person, such as a romantic partner) or was more akin to another form group already reported on (e.g. autistic people or family), the group was not considered as an additional SI.

To determine whether a group acted as *a* social identity for an individual, a similar procedure was used to that of Sani et al. (2015a). If an individual’s average item score within an SI scale was greater than or equal to 5, that group was counted as being *a* social identity for that individual. For SI scales where the average item score was lower than 5 or where appropriate data were not provided, a group was counted as not being a social identity for a participant. A participant’s social identity number (SI Number) was therefore the number of different groups for which they had an average SI score of 5 or greater. Participants could have an SI Number ranging from 0 to 4.

#### Depression: Beck Depression Inventory–Second Edition

The Beck Depression Inventory–Second Edition (BDI-II) is a 21-item self-report measure for assessing the presence of depressive symptoms (Beck et al., 1996). For each item, the participant rates the degree to which they have experienced a symptom in the past 2 weeks, rating on a 4-point scale (0–3). Scores are then summed to give a total depression score, ranging from 0 to 63. Although differences in how depression presents in autistic people may affect how typical depression instruments operate (Stewart et al., 2006), the BDI-II has been found to be the most appropriate existing self-report measure for assessing depression in autistic adults without learning disability (Gotham et al., 2015).

#### Anxiety: State-Trait Inventory for Cognitive and Somatic Anxiety State Measure

The State-Trait Inventory for Cognitive and Somatic Anxiety (STICSA) state measure is a 21-item self-report measure of anxiety (Ree et al., 2000). Participants rate their agreement to each of the statements about anxiety symptoms they are experiencing in the immediate moment on a 4-point scale (1 = Not at all to 4 = Very much so). Item scores are summed to give a total anxiety score, ranging from 21 to 84.

#### Positive Mental Health: Short Warwick–Edinburgh Mental Well-being Scale

The Short Warwick–Edinburgh Mental Well-being Scale (SWEMWBS) is a 7-item measure assessing participants’ positive mental health (Stewart-Brown et al., 2009). Participants rate the amount of time in the past 2 weeks they have experienced a certain thought or feeling on a 5-point scale (1–5, 1 = None of the time, 5 = All of the time). Scores are then summed, giving totals a potential range of 7–35.

### Procedure

The study was administrated through Qualtrics online survey platform. Participants were presented with the information sheet describing the study, their rights to withdraw from the study and that their data would be kept confidentially. Participants indicated their informed consent by selecting an option reading ‘I have read and understood the above and wish to participate in this study’. An option stating a wish to not participate was also provided, and those who selected this were presented with a ‘thank you’ message. Those who indicated consent to the study were then administered the study measures. Following this, the debrief sheet explaining the study and contact information of the researchers was displayed.

## Results

### Pre-analysis checks

Unless otherwise stated, statistical analyses were conducted using SPSS version 25 (IBM Corp., 2017). Prior to parametric tests and regression analyses, total score variables were examined for outliers, multicollinearity and normality. Non-normality was identified in a number of the variables. Square root transformations to correct for positive skew were conducted on BDI and STICSA totals. To correct for negative skew in Autism SI and Family SI scores, square root transformations were conducted on reversed score totals (these were then unreversed such that in the ‘Results’ section, higher scores represent greater feelings of SI). Hereafter, reference to these scores is to the transformed totals.

### Factor structure of the SI scale

The SI scale and its factor structure were examined for reliability through Cronbach’s αs and confirmatory factor analysis (CFA). Responses regarding autism SI and family SI were used for this, as these were answered by the most respondents (n = 184). Cronbach’s αs for the SI scale indicated high internal reliability when assessing autistic SI (α = 0.91) and family SI (α = 0.96). CFA was conducted in R version 3.5.2 (R Core Team, 2018) using the umx package (Bates, 2018). The model of indicator and latent variables as in Leach et al. (2008) was created and then ran using the autism SI item data. Resulting model fit indices are shown in Table 2. Examining absolute fit indices, Hu and Bentler (1999) suggested a root mean square error of approximation (RMSEA) below 0.06 indicates a good fit. More recent opinions suggest cut-offs below 0.07 (Steiger, 2007; notably the deviser of this index) or 0.08 (Hooper et al., 2008). In addition, the standardised root mean square residual (SRMR) is below 0.08, suggesting a good fit. Examining relative fit indices, a Tucker–Lewis index (TLI) and/or a comparative fit index (CFI) greater than 0.95 indicates a good-fitting model relative to the null model and the degrees of freedom. Considering absolute and relative fit indices together suggested the model fit the autism SI data well. The same model of hierarchical factors was run upon the family SI responses. Fit indices suggested this was a poor fit (Table 2).

**Table 2.** Fit indices for social identification models.

	Autism social identification	Family social identification
Absolute fit indices		
RMSEA	0.064^a^	0.115
SRMR	0.047^a^	0.038^a^
Relative fit indices		
CFI	0.967^a^	0.944
TLI	0.957^a^	0.928

RMSEA: root mean square error of approximation; SRMR: standardised root mean square residual; CFI: comparative fit index; TLI: Tucker–Lewis index.

a Suggests a good fit.

## Autistic people’s groups

All 184 participants reported on their SI with other autistic people and their family (see Table 3 for number of participants reporting on each group and subsequently, the number who scored high enough for the group to be considered a social identity). Of these, 94 (51.1%) scored highly enough for other autistic people to be considered as a social identity; 73 (39.7%) scored for their family to be a social identity; 146 (79.4%) answered about a group they had any amount of weekly direct interaction with; 63 (34% of the total sample) scored high enough for their regular contact group to be considered a social identity. Most reported on their feelings towards colleagues or customers at work. College peers, autism support groups and social groups were also common. However, 30 (16.3%) did not report having any group of people they had regular direct interaction with. 59 (32.1%) self-nominated a group and reported their feelings of SI towards it. 42 of these (22.9% of total sample) had an average SI item score greater than 5, and the nominated group was considered a social identity. Summing together the number of groups participants socially identified with, approximately one-quarter of the sample (24.5%) reported not socially identifying with any groups, whereas roughly one-fifth had three or four social identities (19%; see Table 4).

**Table 3.** Social identities.

Group	Reported on group		Group as social identity	
	n	% of total sample	n	% of total sample
Other autistic people	184	100	94	51.1
Family	184	100	73	39.7
Regular contact group	146	79.4	63	34
Other self-nominated group	59	32.1	42	22.9

**Table 4.** Numbers of social identities (N = 184).

No. of social identities	n	%
0	45	24.5
1	49	26.6
2	55	29.9
3	27	14.7
4	8	4.3

## Descriptive statistics and correlations

Descriptive data and correlations of variables are shown in Table 5 (prior to any transformations). For Contact SI, 146 participants answered about a regular contact group. For Self-Nom SI, 59 answered about an appropriate group. Thus, descriptive statistics and correlations for Contact SI and Self-Nom SI were calculated using only data from these participants while accepting the possibility of Type II error due to being underpowered for these groups. There were no significant gender differences in SRS (*F*(2, 181) = 0.16, p = 0.85) or well-being scores (depression: *F*(2, 181) = 0.31, p = 0.74; anxiety: *F*(2, 181) = 0.87, p = 0.42; positive mental health: *F*(2, 181) = 0.87, p = 0.87). There were no significant differences between those with and without a formal diagnosis on SRS scores (t = 0.25, df = 182, p = 0.80) or on well-being scores (depression: t = 0.56, df = 182, p = 0.58; anxiety: t = 0.32, df = 182, p = 0.75; positive mental health: t = 0.30, df = 182, p = 0.76).

**Table 5.** Descriptive statistics and correlations (N = 184).

	*M*	*SD*	1	2	3	4	5	6	7	8
1. Age (years)	41.0	12.7	–							
2. Autistic traits	110.0	26.9	0.07	–						
3. Depression	21.3	12.8	0.14	0.45**	–					
4. Anxiety	40.0	12.6	0.11	0.40**	0.70**	–				
5. Positive mental health	19.9	4.8	−0.22**	−0.41**	−0.74**	−0.48**	–			
6. Autism SI	68.8	15.1	−0.04	0.00	−0.29**	−0.03	0.31**	–		
7. Family SI	61.5	23.1	−0.10	−0.23**	−0.29**	−0.20**	0.27**	0.32**	–	
8. Contact SI (n = 146)	64.3	19.2	−0.08	−0.19*	−0.08	0.12	0.25**	0.34**	0.16	–
9. Self-nom SI (n = 59)	75.3	16.2	0.18	−0.04	−0.07	0.04	0.07	0.32*	0.01	0.16

SD: standard deviation; SI: social identification.

* p<0.05, **p<0.01

## SI and mental well-being

Multiple linear regression models were conducted to examine whether autism and family SI were predictors of each of depression, anxiety and positive mental health. Gender, Age, SRS Scores, Autism SI and Family SI were the independent variables in all three analyses. As gender had three nominal categories (male, female and other), two dummy variables were created. Female gender was set as the baseline group. β values and semi-partial correlations squared to indicate independent variable effects are shown in Table 6.

**Table 6.** Social identification types and mental well-being (N = 184).

Predictor	Depression		Anxiety		Positive mental health	
	β	r^2^_(x,y)_	β	r^2^_(x,y)_	β	r^2^_(x,y)_
Gender – male	−0.04	0.001	−0.04	0.001	−0.00	0.000
Gender – other	−0.06	0.003	−0.10	0.012	−0.04	0.002
Age	0.11	0.012	0.05	0.003	−0.19**	0.045
Autistic traits	0.43***	0.176	0.38***	0.146	−0.38***	0.163
Autism social identification	−0.22**	0.048	0.01	0.000	0.25***	0.074
Family social identification	−0.12	0.014	−0.10	0.009	0.09	0.010

** p < 0.01, ***p < 0.001.

For depression, the variables together predicted a significant amount of variance (*F*(6, 177) = 13.01, p < 0.001, adjusted R^2^ = 0.28, f^2^ = 0.09). Autistic trait scores (β = 0.43, semi-partial r^2^ = 0.18) and autism SI (β = −0.22, semi-partial r^2^ = 0.05) were found to be significant individual predictors of depression (see Table 6). Standard statistics and plots suggested regression assumptions were met and the model’s parameters were generalisable to other samples.

For anxiety, the regression model was significant (*F*(6, 177) = 6.72, p < 0.001, adjusted R^2^ = 0.16, f^2^ = 0.03). However, autistic trait scores (β = 0.38, semi-partial r^2^ = 0.15) were the only significant individual predictor of anxiety (Table 6). Regression assumptions were met.

For positive mental health, the regression model was significant (*F*(6, 177) = 12.20, p < 0.001, adjusted R^2^ = 0.27, f^2^ = 0.08). Age (β = −0.19, semi-partial r^2^ = 0.05), autistic trait scores (β = −0.38, semi-partial r^2^ = 0.16) and Autism SI (β = 0.25, semi-partial r^2^ = 0.07) were each significant individual predictors of positive mental health (Table 6). Statistics and plots indicated that the model’s parameters were generalisable to other samples.

## Number of social identities and depression

Multiple linear regression analyses were used to examine whether the number of social identities participants had predicted their mental well-being scores. Gender, Age, SRS scores, Autism SI and Family SI were the independent variables in all three analyses. As gender had three nominal categories (male, female and other), two dummy variables were created. Female gender was set as the baseline group. β values and semi-partial correlations squared to indicate independent variable effects are shown in Table 7.

**Table 7.** Number of social identities and mental well-being (N = 184).

Predictor	Depression		Anxiety		Positive mental health	
	β	r^2^_(x,y)_	β	r^2^_(x,y)_	β	r^2^_(x,y)_
Gender – male	−0.02	0.001	−0.01	0.000	0.00	0.000
Gender – other	−0.06	0.003	−0.09	0.009	−0.03	0.001
Age	0.11	0.011	0.06	0.004	−0.18**	0.039
Autistic traits	0.43***	0.188	0.41***	0.163	−0.37***	0.154
SI number	−0.19**	0.036	0.02	0.001	0.25***	0.075

SI: social identification.

** p < 0.01, ***p < 0.001.

The regression model for depression predicted a significant amount of the variance (*F*(5, 178) = 12.83, p < 0.001, adjusted R^2^ = 0.24, f^2^ = 0.32). Of the independent variables, autistic traits (β = 0.43, semi-partial r^2^ = 0.19) and SI Number (β = −0.19, semi-partial r^2^ = 0.04) were the only significant predictors of depression scores. Standardised residuals revealed the model to be accurate across the sample. Linear regression assumptions were also met, indicating the generalisability of the model.

The regression model predicting anxiety scores was found to be significant (*F*(5, 178) = 7.71, adjusted R^2^ = 0.16, p < 0.001, f^2^ = 0.19). However, the only significant predictor in the model was autistic traits (β = 0.41, semi-partial r^2^ = 0.16). Statistics and plots suggested the model was accurate and generalisable.

A significant amount of variance in positive mental health scores was predicted by its model (*F*(5, 178) = 13.02, adjusted R^2^ = 0.25, p < 0.001, f^2^ = 0.33). Age (β = −0.18, semi-partial r^2^ = 0.04), autistic traits (β = −0.37, semi-partial r^2^ = 0.15) and SI Number (β = 0.25, semi-partial r^2^ = 0.08) were significant predictors. A number of statistics indicated that data from participants of other gender may have been influencing the model. However, a regression model without other gender participants (and without the other gender dummy variable) was no more accurate. Thus, the original model was retained. Statistics and plots suggested this model would be generalisable to other samples.

## Discussion

This study explored autistic adults’ SI with a range of different groups, and whether these feelings were associated with mental well-being. A previously suggested factor model of SI appeared to apply to autistic adults when asked to consider other autistic people, but not when considering their family. Autistic adults were found to socially identify with many kinds of groups, including other autistic people, their family, work and peer groups and hobby groups. Some participants socially identified with multiple groups, whereas some did not socially identify with any group. More strongly socially identifying as autistic, with one’s family and with more groups overall was associated with less depression and greater positive mental health.

Previous theories have described autistic people as being distinct from neurotypical people in many social domains, such as having less motivation for social relationships (Chevallier et al., 2012) and obtaining less pleasure from social interaction (Han et al., 2019). In contrast to this, the current study found that autistic adults broadly understood questions regarding SI similarly to non-autistic people. This indicates that autistic experiences of some aspects of social relationships may be similar to those of the rest of the population. However, the hierarchical model did not apply to autistic adults’ SI with their family. To the researchers’ knowledge, this model of factors has not been examined in relation to family SI before in autistic nor non-autistic samples. The data suggested that SI may not operate in quite the same manner for family groups as other groups. Beyond personal and social identities, some have also proffered the importance of self-definition in terms of relational identities, identities derived from dyadic relationships (Brewer & Gardner, 1996). It is possible that self may be perceived more in terms of these interpersonal role-relationships (e.g. mother and son), rather than perceiving the family as a distinct collective unit (e.g. the Smith family). Thus, relational identification processes, through which the self is determined in terms of role-relationships, may predominate over SI processes, where the self is defined in terms of a group’s collective characteristics (Sluss & Ashforth, 2007).

Autistic adults reported feeling SI with various groups. The most common social identity was being an autistic person, followed by the family group. When given the option of reporting on any additional group they had contact with, 23% identified such a group that operated as a social identity for them. Considering the number of groups participants socially identified with, 19% indicated having three or four groups with which they felt connected. This adds to mounting evidence that counter to the social motivation hypothesis (Chevallier et al., 2012), many autistic people do desire and form social relationships (see Jaswal and Akhtar (2019) for a commentary on this evidence). However, approximately one-quarter did not have any group with which they felt a strong degree of SI. This is substantially more than in studies with general population samples, such as 5% in Sani et al. (2015b) and 7% in Miller et al. (2015). This is consistent with higher levels of loneliness reported by autistic adults (Mazurek, 2014). However, the range in number of social identities in the sample also coincides with recent positions on social motivation, proposing wide individual differences across the autistic population (Mundy, 2019). Together, the current results show that there is high variation in the types and numbers of groups that autistic people are a part of and feel connected to, but on average they have fewer group connections than typically found.

Socially identifying as autistic was found to associate with lower depression and higher positive mental health. This is consistent with the findings of Cooper et al. (2017), who found that autism SI was related to depression through its association with collective and personal self-esteem. However, unlike Cooper et al. (2017), this study did not find an association between autism SI and anxiety. This difference may be due to a difference in measures – Cooper et al. (2017) used a trait measure of anxiety, whereas this study used a state measure. It appears that an enduring tendency to feel anxious may be related to social identifying as autistic, but immediate feelings of anxiety are not. This could be explained as SI itself being a more enduring variable rather than a state variable (Cruwys et al., 2014; McGarty, 2001). Alternatively, this could be related to power, as it could be expected that there would be a smaller effect with state versus trait measures (Epstein, 1979).

Family SI was associated with lower depression and anxiety, and higher positive mental health scores. This is consistent with findings in general population samples, with higher SI with family associated with lower depression and stress and greater life satisfaction (Sani et al., 2012). However, given that the SI scale’s factor structure did not fit for this autistic sample, these associations may not definitively be with SI as we understand it.

Finally, socially identifying with a higher number of groups was associated with lower depression and higher positive mental health. This is consistent with work in the general population (Cientanni et al., 2017; Miller et al., 2015; Sani et al., 2015b) and suggests that the ‘more-the-merrier’ theory, whereby social identity benefits for mental well-being are additive across multiple groups (Iyer et al., 2009), is applicable to autistic adults. However, each of the semi-partial correlations of a SI variable with a mental health outcome variable were low (< 0.1). This indicates that SI has little independent effect upon mental well-being, and that other variables are involved in relationships between the two.

Autistic traits were the strongest predictor of mental well-being in all regression models. Autistic behaviours have been found to associate with poorer mental well-being through negative cognitive patterns, such as attributional style (Barnhill & Smith Myles, 2001; Farrugia & Hudson, 2006), rumination (Gotham et al., 2014; Patel et al., 2017) and social problem-solving ability (Jackson & Dritschel, 2016; Rosbrook & Whittingham, 2010). The current results indicate the need to elucidate exactly how autistic traits associate with poorer mental well-being.

### Implications

Interventions exist which foster SI. Social prescribing is a health-based recommendation for individuals to join community groups to reduce loneliness and improve mental well-being (Royal College of General Practitioners, 2018). More specifically targeting SI, the Groups 4 Health programme is a group psychoeducational intervention designed for the general population (Haslam et al., 2016). It has been found to help participants feel more connected to multiple groups, and has been associated with improvements in mental well-being. Such interventions may be similarly helpful for improving mental well-being in autistic people specifically. The potential benefits of social prescribing for autistic people’s mental well-being have recently been explored (Charlton et al., 2020). However, autistic social differences may need to be taken into account by such interventions, such as differences in emotion processing and perspective-taking (Ghanouni et al., 2019; Orsmond et al., 2004), intensity of interests (Ghanouni et al., 2019) and sensory sensitivities (Reynolds et al., 2011; Wang & Berg, 2014). Individuals, both autistic and non-autistic, may need to adapt their interaction in order to be part of the same groups. In addition, the potential decreased tendency to self-categorise into social groups may also limit social integration and identification in autistic adults (Skorich et al., 2017; Skorich et al., 2016). Alternative to this focus on the individual, there are societal factors which may prevent autistic people from being part of social groups. Autistic people can face misunderstanding, stigma and bullying from others. Non-autistic people have been found to be poor at interpreting the thoughts and feelings of autistic people (Sheppard et al., 2016), to underestimate autistic people’s ability to consider other’s perspectives (Heasman & Gillespie, 2018) and show unwillingness to interact and spend time with autistic people (Sasson et al., 2017). Studies have found high rates of victimisation among autistic people, with observed rates ranging from 40% to 96% (Humphrey & Hebron, 2014). Together this suggests that many neurotypical-dominant groups may not be accepting of autistic people. Providing autistic people with opportunities to engage with other autistic people may be advantageous as there will be fewer barriers to engagement than in non-autistic groups as discussed. Autistic adults report significantly fewer difficulties in interacting with other autistic people compared to when interacting with non-autistic people (Gernsbacher et al., 2017). Information communication along chains of autistic individuals has been shown to be as effective as along chains of non-autistic individuals, and interactional rapport is as good in the autistic chains as in non-autistic chains (Crompton et al., 2020). This means it may be easier to promote autistic adults’ feelings of SI with autistic groups compared to other groups.

### Strengths and limitations

One strength of the study was the inclusion of self-diagnosed autistic individuals, if they met the cut-off criteria for level of autistic traits. Including self-diagnosed autistics enabled a larger sample and allowed the examination of whether these participants differed from those with a formal diagnosis (which they did not appear to). Another strength was giving participants the option to self-nominate a group to report upon, as this means the study was more likely to capture idiosyncratic groups that people can belong to. One possible limitation is that the sample predominantly consists of females (62.5%) who are in full-time employment (35.9%) have at least a bachelor’s degree (67.4%) and are married or living with a long-term partner (47.3%). Results may potentially be representative of effects in adults of these demographics only. In addition, participation required independent use of the Internet and completion of measures. The present results should therefore not be generalised to autistic adults with co-occurring conditions which may mean they cannot independently fill out self-report measures online (e.g. Intellectual Disability, dementia). Other limitations of the study include its cross-sectional nature, in that no conclusions can be drawn about directionality, and the lack of a non-autistic group to allow direct comparisons to be made. In addition, the measures of mental health used were not specifically tailored for autistic people, and given that autistic people may experience and report on their mental health differently to non-autistic people, typical measures may not be wholly accurate (Kerns et al., 2014; Wigham et al., 2017). Finally, in asking participants to self-nominate a group after answering questions about their family and regular contact group, they may have only considered groups that they have some form of direct interaction with. They may not have considered wider collective groups, such as those of nationality, race and so on. As such, providing examples of such potential social identity groups may have meant more participants reporting a self-nominated social identity.

## Conclusion

This study found that autistic adults experience and report on feelings of SI with groups in a similar way to non-autistic people. Feeling connection to more groups was associated with lower depression and more positive mental health. Specifically, socially identifying with other autistic people and one’s family was associated with better mental health outcomes. This illustrates the importance of enabling autistic people to participate in social groups, but with other autistic people in particular.
